# Failure of anti Interleukin-1 β monoclonal antibody in the treatment of recurrent pericarditis in two children

**DOI:** 10.1186/s12969-020-00438-5

**Published:** 2020-06-16

**Authors:** Sara Signa, Matteo D’Alessandro, Rita Consolini, Angela Miniaci, Marta Bustaffa, Chiara Longo, Maria A. Tosca, Martina Bizzi, Roberta Caorsi, Leonardo Oliveira Mendonça, Andrea Pession, Angelo Ravelli, Marco Gattorno

**Affiliations:** 1 UOSD Centro Malattie Autoinfiammatorie ed Immunodeficienze, IRCCS Istituto Giannina Gaslini, Via Gerolamo Gaslini 5, 16147 Genoa, Italy; 2 DINOGMI, Università di Genova, Genoa, Italy; 3 Clinica pediatrica, Università di Pisa, Pisa, Italy; 4 Pediatric Unit, Department of Medical and Surgical Sciences, S. Orsola Hospital, University of Bologna, Bologna, Italy; 5 UOSD Centro Allergologia IRCCS Istituto Giannina Gaslini, Genoa, Italy; 6 Clinica Pediatrica e Reumatologia, IRCCS Istituto Giannina Gaslini, Genoa, Italy

**Keywords:** Recurrent pericarditis, Interleukin-1 β, Interleukin-1 α, Anakinra, Canakinumab

## Abstract

**Background:**

Recurrent pericarditis (RP) is a complication (15–30%) of acute pericarditis with an unknown etiology. Treatment regimen consists of a combination of non-steroidal anti-inflammatory drugs (NSAIDs) and colchicine, with the addition of corticosteroids in resistant or intolerant cases. In the last decade anakinra was shown as an effective treatment in patients with colchicine resistant and steroid-dependent RP, initially in anecdotal reports in children and more recently in a randomized trial. Canakinumab is a monoclonal antibody selectively blocking IL-1β and its use is only anecdotally reported to treat pericarditis. We report two pediatric patients with refractory recurrent pericarditis, who presented an optimal response to anakinra treatment but prompt relapse after switch to canakinumab.

**Case presentation:**

The first patient is a girl with Recurrent Pericarditis started in April 2015, after heart surgery. NSAIDs and oral steroids were started, with prompt relapse after steroid suspension. The child showed a steroid-dependent RP; anakinra was therefore started with excellent response, but discontinued after 2 weeks for local reactions. In July 2016 therapy with canakinumab was started. She experienced four relapses during canakinumab therapy despite dosage increase and steroid treatment. In January 2018 a procedure of desensitization from anakinra was performed, successfully. Anakinra as monotherapy is currently ongoing, without any sign of flare.

The second patient is a girl with an idiopathic RP, who showed an initial benefit from NSAIDs and colchicine. However, 10 days after the first episode a relapse occurred and therapy with anakinra was established. Two months later, while being in complete remission, anakinra was replaced with canakinumab due to patient’s poor compliance to daily injections. She experienced a relapse requiring steroids 10 days after the first canakinumab injection. Anakinra was subsequently re-started with complete remission, persisting after 24 months follow-up.

**Conclusions:**

We describe two cases of failure of the treatment with anti-IL-1β monoclonal antibodies in steroid- dependent idiopathic RP. This anecdotal and preliminary observation suggests a different efficacy of the two IL-1 blockers in the management of RP and support a possible pivotal role of IL-1α in the pathogenesis of this condition.

## Background

Recurrent pericarditis (RP) is a complication (15–30%) of acute pericarditis with an unknown etiology [1]. Treatment regimen consists of a combination of non-steroidal anti-inflammatory drugs with colchicine with the addition of corticosteroids in resistant or intolerant cases [2]. Anakinra is a therapeutic option as steroid-sparing agent, as recently demonstrated by the first anecdotal reports in children and adults [2–4] and corroborated by the recent AIRTRIP trial [5] and IRAP study [6]. Anakinra neutralizes the biologic activity of interleukin-1α (IL-1α) and interleukin-1β (IL-1β) by inhibiting their binding to IL-1 type I receptor. Another IL-1 blocker is canakinumab, a monoclonal antibody that selectively blocks IL-1β in circulation. Canakinumab treatment in pericarditis has been reported only anecdotally [7, 8] and its effectiveness in treating this clinical manifestation need to be further inquired and validated.

We report our experience concerning Canakinumab in two pediatric patients suffering from refractory recurrent pericarditis, who presented an optimal response to anakinra treatment but prompt relapse after the canakinumab therapy start. One patient needed desensitization procedure to anakinra to reintroduce the treatment and achieve clinical remission.

## Case presentation

**Fig. 1:**
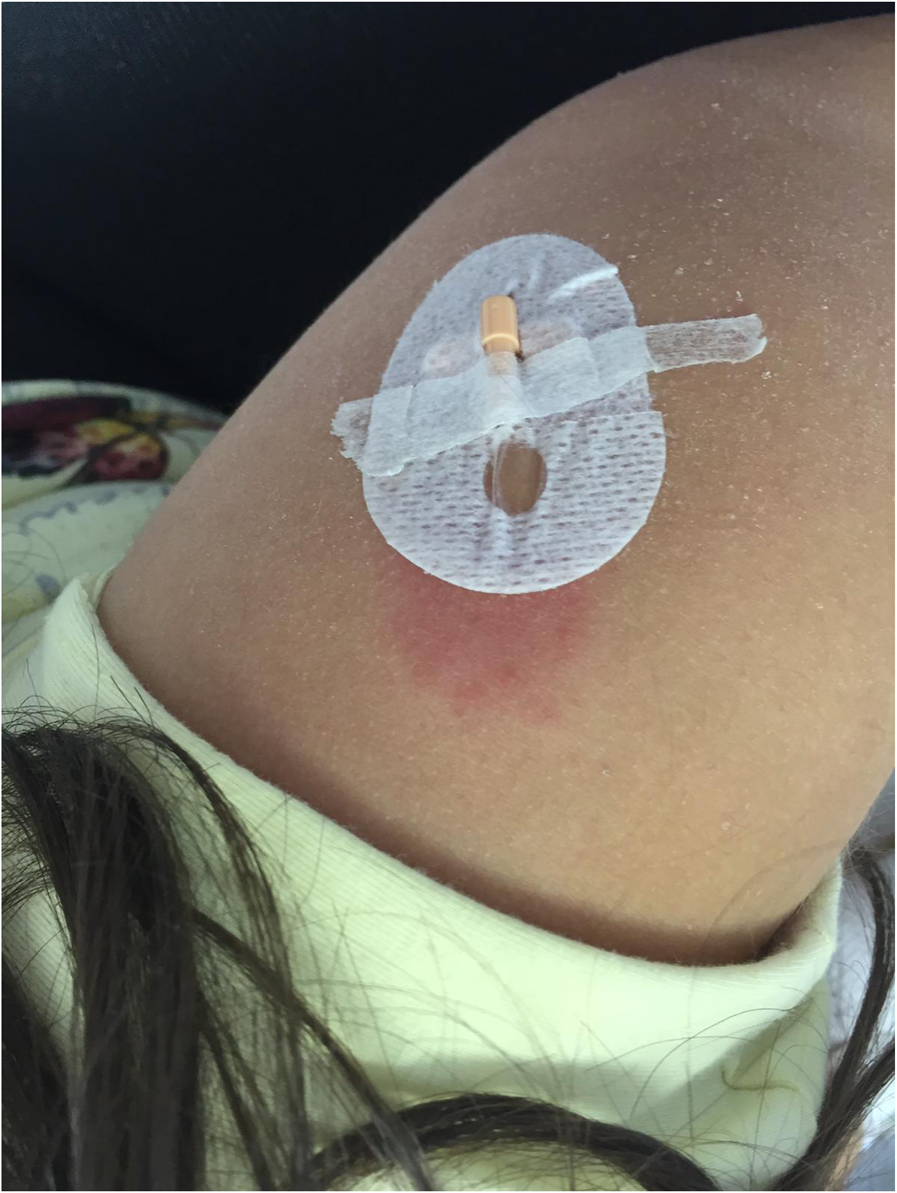
Local erythema and edema at anakinra injection site in patient 1

**Fig. 2:**
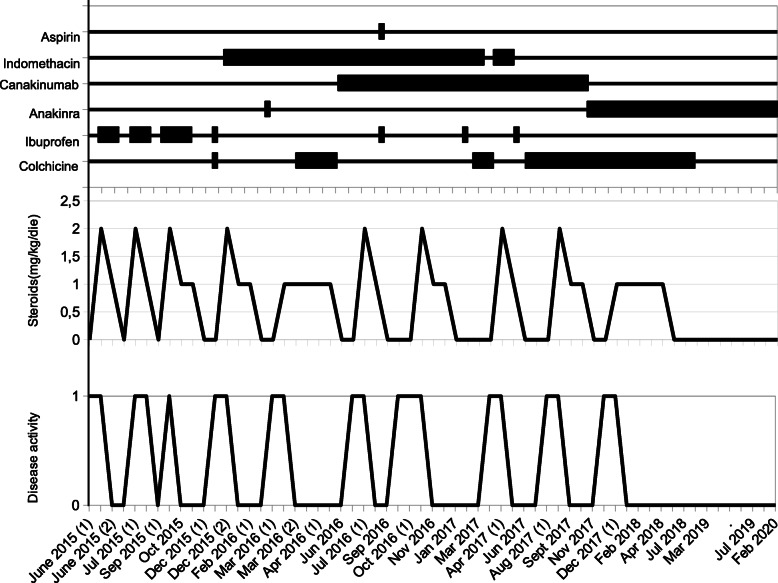
*Therapy and clinical activity details.* Treatment regimens, steroid therapy dosage and disease activity (0 = inactive; 1 = active) during the disease course in patient 1

Patient 1 is a 10-years old girl, admitted to our hospital in 2016 because of RP started in June 2015, 2 months after a surgical correction for atrial septal defect. Non steroidal anti-inflammatory drugs (NSAIDs), colchicine and oral steroids were started and then gradually tapered, with a disease flare after the steroid suspension, requiring pericardiocentesis. The child showed a steroid-dependent RP, with several relapses at steroid tapering. In March 2016, after five relapses, anakinra (2 mg/kg/day) was started, with a fast and complete clinical response. However, it was discontinued after 2 weeks for the appearance of a diffuse itchy urticarial rash and a delayed local response with swelling and erythema at the injection site, unresponsive to antihistamines. Due to the persistence of pericarditis relapses and steroid dependency despite colchicine treatment, in July 2016 canakinumab was started (4 mg/kg every 4 weeks) with NSAIDs and steroids, that were subsequently gradually tapered. During the following 6 months, the patient presented four relapses at any attempt to reduce the steroids below 0.25 mg/kg/die. The modification of the schedule of canakinumab administration to 4 mg/kg every 3 weeks and the reintroduction of Colchicine were not beneficial. Therefore, in December 2017 canakinumab was withdrawn. Due to the severe steroid toxicity, we decided to attempt the reintroduction of anakinra after desensitization process, as previously reported in other patients with IL-1-mediated conditions [9] and summarized by Yilmaz [10]. The desensitization started in January 2018 with five to three consecutive injections per day of gradually increasing anakinra doses and dilutions from days 1 to 9 (Table 1). Each injection was spaced by 15 min intervals, raising the dose at each step. On Day 2, due to the appearance of skin reactions at injection site, the interval between the injections was increased to 30 min with increased dilution, restarting the desensitization protocol. The full target dosage (80 mg/day; 2 mg/kg/day) at standard dilution (divided in 4 different administrations) was reached on Day 8. Since Day 11 anakinra was administrated twice a day. Antihistaminic and steroids were administrated during all the desensitization process and extremely slowly tapered in the subsequent 6 months due to the persistence and recurrence of local erythema and edema at the injection site (Fig. 1). Anakinra was finally administrated once a day since June 2018. Antihistamines and steroids were definitively stopped, without recurrence of either skin reactions or disease flares. In June 2018, low-dose colchicine was progressively tapered and finally discontinued. After 24 months follow-up, the patient is still on daily anakinra as monotherapy. Neither flares of pericarditis nor injections skin reactions were observed anymore (Fig. 2).

**Table 1:** *Desensitization to anakinra schedule.* Desensitization pre medication: cetirizine 10 mg twice a day and oral metilprednisolone 4 mg twice a day

	TIME INTERVAL (minutes)	DOSE (mg)	DILUTION (mg/mL)
**Day 1**	0	0,1	1
	15	0,3	1
	30	0,6	1
	45	1	5
	60	2,5	5
**Day 2**	0	2,5	5
	30	3,75	5
	60	5	5
	90	6,25	10
	120	7,5	10
**Day 3**	0	7,5	10
	30	8,75	10
	60	10	20
	90	12,5	20
**Day 4**	0	8,75	10
	30	10	10
	60	12,5	20
**Day 5**	0	12,5	20
	30	15	20
	60	17,5	20
**Day 6**	0	17,5	40
	30	20	40
	60	22,5	40
**Day 7**	0	22,5	40
	30	25	40
	60	30	40
**Day 8**	0	30	40
	30	20	40
	60	30	40
**Day 9**	0	30	60
	30	20	60
	60	30	60
**Day 10**	0	30	149
	30	20	149
	60	30	149
**Day 11**	0	40	149
	30	40	149

Patient 2 is an 11-years-old girl with idiopathic RP, diagnosed in April 2017, requiring a pericardiocentesis at disease onset. She initially benefit from NSAIDs and colchicine. However, 10 days after the first episode a relapse occurred; anakinra was therefore started with a dramatic and complete response. Two months later, while the patient was in in complete remission, anakinra was replaced with canakinumab (2.5 mg/kg/dose) due to poor compliance to daily injections. Ten days after the first canakinumab injection she experienced a severe relapse requiring oral steroids. Anakinra (2 mg/kg/day) was re-started allowing fast steroid tapering. She showed complete remission in anakinra as monotherapy, persisting after 26 months follow-up.

## Discussion and conclusions

We describe two cases of substantial treatment failure with anti-IL-1 β monoclonal antibodies treatment in RP, one idiopathic and one post-pericardiotomy. In both, a good response to recombinant IL-1 receptor antagonist as monotherapy was achieved. Notably, while canakinumab is selectively targeting IL-1 β ▢ anakinra prevents the biological activity of both IL-1 α and IL- β. The active IL-1 β is secreted by monocytes and macrophages the activation of the Inflammasomes. Conversely, IL-1α is constitutively expressed in several types of cells at steady state, especially in epithelial cells, activated by cellular stress and massively secreted after cell necrosis [11].

The desensitization from anakinra in patient 1 was rather long and laborious. The timing and type of reaction suggest a mixed IgE and non IgE mediated mechanisms, an justify the longer period needed to achieve a complete result than previously described in the literature [10]. Nonetheless, the process allowed the complete control of disease flares with a relevant impact on patient’s quality of life.

An anecdotal observation [7] of good answer to canakinumab in two Adult-onset Still’s Disease patients with pericarditis was reported, whereas a third patient with seronegative RA relapsed, requiring steroid therapy. In pediatric patients a case of a child with idiopathic RP with anaphylactic reaction to anakinra was recently described [8]. In this case very high doses (5 mg/kg monthly) of canakinumab were able to maintain the clinical remission in association with colchicine. While all data so far available in the literature show the possibility to obtain complete response with anakinra in RP, at least when used as the scheduled daily regimen [1, 5, 12], the present report suggest that anti-IL-1 β monoclonal antibody may have a less clear impact in the treatment of this condition. This might support the relevance of IL-1 α in the induction and maintenance of the inflammatory response at the tissue level in idiopathic pericarditis [11].

In conclusion, we describe two cases of substantial failure of the treatment with anti-IL-1β monoclonal antibodies treatment in steroid- dependent idiopathic RP. In both case a good response to the treatment with recombinant IL-1 receptor antagonist was achieved. These anecdotal and preliminary observations suggest a different efficacy of the two IL-1 blockers in the management of recurrent pericarditis and may stimulate further inquiries on the role of IL-1 (particularly IL-1α) in the induction and maintenance of the inflammatory response at the tissue level in idiopathic pericarditis.

## Data Availability

Not applicable.

## Abbreviations

IL-1: Interleukin-1
IL-1α: Interleukin-1α
IL-1β: Interleukin-1β
NSAIDs: Nonsteroidal anti-inflammatory drugs
RP: Recurrent pericarditis
